# Road traffic injuries in China from 2007 to 2016: the epidemiological characteristics, trends and influencing factors

**DOI:** 10.7717/peerj.7423

**Published:** 2019-08-06

**Authors:** Xue Wang, Huiting Yu, Chan Nie, Yanna Zhou, Haiyan Wang, Xiuquan Shi

**Affiliations:** 1 Department of Epidemiology and Health Statistics, School of Public Health, Zunyi Medical University, Zunyi, Guizhou, China; 2 Center for Injury Research and Policy & Center for Pediatric Trauma Research, The Research Institute at Nationwide Children’s Hospital, The Ohio State University College of Medicine, Columbus, OH, USA

**Keywords:** Traffic accidents, RTIs, Areas, Road surface

## Abstract

**Background:**

Road traffic accidents are one of the serious disasters that cause public injury, fatality and great economic loss. They are a growing public health problem around the world.

**Objectives:**

The aim of this study was to determine epidemiological characteristics, tendency and possible influencing factors of road traffic injuries (RTIs) in China, so as to give target suggestions on preventative measures.

**Methods:**

Road traffic accident data were obtained from National Bureau of Statistics of China and Ministry of Transport of the People’s Republic of China. Descriptive statistic such as RTIs frequency, trends of different accident types from 2007 to 2016; the RTIs difference between different regions and road surfaces were compared; and the possible influencing factors of RTIs were also explored.

**Results:**

Over the past decade, with the mileage of constructed highway increased, the frequency of road traffic accidents have declined substantially in China, and the death toll from road traffic accidents with motor vehicles has declined from 2007 to 2015, Conversely, the number of deaths from non-motor vehicle accidents has risen rapidly since 2012. Our study showed that the traffic accident related mortality in Guizhou province was different from the level of the whole nation, and the Eastern, Central and Western areas of China were all significantly different (*P* < 0.001). Linear regression suggested a significant affected of gross domestic product (GDP)-per-capita, education level, the number of health institutions, populations, and car ownership status on traffic accident death tolls (*P* < 0.001). Moreover, cement concrete pavement roads were associated with the highest occurrence rates of RTI, and RTIs was statistically significant (*P* < 0.001) on different road surfaces.

**Conclusion:**

Even though the frequency of road traffic accidents has declined, RTIs remain an urgent public health problem in China. Thus, the government should give some target preventative measures to reduce RTIs, aiming at different regions, the increasing trend of the death toll related to non-motor vehicles and the highest occurrence on cement concrete pavement roads.

## Introduction

Road traffic injuries (RTIs) are the main causes of death, hospitalization, disability, and direct economic loss around the world. The World Health Organization (WHO) reports that 90% of the world’s fatalities on the road occur in low- and middle-income countries. These countries have more than half of the world’s vehicles and WHO states that approximately 1.35 million people die each year as a result of road traffic crashes (WHO, 2018). From 2010 to 2013, developed countries had a 2.0–4.6 times larger decrease in both overall and user-specific road mortality compared to developing countries (Ning et al., 2016). WHO estimated one fifth of road traffic deaths occur in China (WHO, 2015). Compared to high-income countries, road traffic accidents are increasing in low- and middle-income countries every year. High-income countries were found to have a lower total number of deaths than low- and middle-income countries, possibly due to those countries maintaining higher vehicle standards and policies (Pal et al., 2018).

It was reported that RTIs occurred in 2.2% of the population and accounted for 12.9% of all injuries. Some people predict that the number of deaths and years of life lost (YLL) from road traffic crashes will decrease slightly, however the number of deaths and YLL is also predicted to increase from 2015 to 2030 due to RTIs (Tan et al., 2014a; Tan et al., 2014b). In 2009, road traffic deaths accounted for about 70,000 premature deaths in China and road traffic accidents disproportionately affected the following populations: males, persons 21–65 years old, adults aged more than 65 years, persons living in rural areas, pedestrians, passengers, motorcyclists and bicyclists (Zhang et al., 2013). Rapid economic growth and intensive motorization accompanied rising rates of traffic fatalities (Blumenberg et al., 2017; Feng et al., 2014), and one of the main factors contributing to traffic fatalities is the growing number of motor vehicle. It is estimated that about 55 thousand new motor vehicles are registered in China every day. From 1996 to 2015, the motorization rate showed rapid growth, increasing from 0.023 to 0.188 (/Person) (Wang et al., 2018). Although China has significantly higher rates of mortality and lager hospitalization burdens of RTIs than high-income countries, it has done little to provide road safety statistics and to conduct road safety researches, so RTIs are still a largely neglected public health problem.

As the biggest developing country with wide territories, the expansion of road networks and surges in personal vehicle ownership are having profound effects on public health in China. China is also a high-density population and a newly motorized country, its traffic accident-related situation is different from other countries. Although major patterns of traffic crashes in China have been reported (Zhang et al., 2013) and a few previous studies offer some data for Chinese road traffic safety trends and disparities in road traffic mortality based on sociodemographic factors, geographical regions, and types of road user used police-reported data from 1985 to 2005 and from 1951 to 2008 (Hu et al., 2008; Zhang et al., 2011). It still lacks adequate data about traffic accidents in different classified vehicles, regions, and road surface types. Thus, it is necessary to offer these details in the RTIs epidemiology research.

In March 2016, the Chinese government approved its 13th Five-Year Plan for National Economic and Social Development (2016 to 2020). The goal on the wellbeing of the plan is to increase one year of the life expectancy in the next five years. To make a contribution to achieve this goal, reducing RTIs should be an important way. Only when we gain a clear understanding of the historical situation can we do a good job of prevention in the future. Thus, this study reported the details of road traffic accidents in China from 2007 to 2016. We want to test the hypotheses that different areas and different types of road surfaces have different RTIs in China. We aim to reveal the trends and possible influencing factors for RTIs, and give some target preventative suggestion for policy makers in China, even for some other similar developing countries besides China.

## Methods

### Data Source

This longitudinal study was based on national data. Data were extracted from National Bureau of Statistics of China (http://www.stats.gov.cn/) and Ministry of Transport of the People’s Republic of China (http://www.mot.gov.cn/) for the time period from 2007 to 2016. The information includes the basic road traffic accident characteristics (e.g., road traffic frequency, death toll, and number of injured people, and economic loss). Population data, vehicles data, gross domestic product (GDP)-per-capita, education level, health institutions data, car ownership status, road surface types were obtained from the Annual Statistics Communique and Statistical Yearbook. In order to ensure the quality of the collected data, any duplicate or redundant information concerning the injuries was cleaned. The data are updated every two years, so 2016 was the latest year we had access to. Data used in this study related to road accidents and possible influencing factors were all collected from the above official websites. Our analyses excluded the data of Taiwan, Hong Kong, and  Macau because the data of these areas are not available.

### The inclusion/exclusion criteria and study design

The data included in this study had to be available, complete, and contain the road traffic accident frequency, death toll, quantity of injured people, direct economic loss, and possible influencing factors for the accident. Data was excluded if it was from more than ten years ago or if some main data fields were missing. A cross-sectional study design was used in our study.

### Definition of study variables

(1) According to the inclusion criteria of the traffic police departments around China, road traffic accident categories are defined to include all traffic related crashes (motor vehicle traffic accidents, non-motor vehicle traffic accidents, pedestrian and passenger related traffic accidents, and the ‘other’ traffic category).
(2) Road traffic accident fatalities are defined to include all traffic-related deaths that occur within 7 days after the crash event.
(3) Accident regions are classified into three groups: Eastern region (economically developed coastal areas), Western region (the less-developed interior areas), and Central region (the middle economies, between Eastern and Western regions).
(4) Independent variables in multiple linear regression models explaining the number of deaths included the following independent variables: GDP-per-capita, education level (Graduates above primary school), the number of health institutions (One of the main indicators of medical status which include basic health-care facilities), population, and car ownership status. Related definitions can be found from the website of National Bureau of Statistics of China (http://www.stats.gov.cn/english/ClassificationsMethods/Definitions/).
(5) According to the inclusion criteria of Technical Standard of Highway Engineering (2003), road surface types were classified into four groups: asphalt concrete pavement, cement concrete pavement, simple pavement, and unpaved roads.

### Statistical analysis

Annually frequency of road traffic accidents were used as the basic variable to conduct statistics and analysis. Descriptive epidemiology was used to describe the characteristics of RTIs. Microsoft Office Excel (version 2010) was used to build the database and SPSS (version 18.0, IBM Corp., Armonk, NY, USA) was used for data analysis. Chi-square tests were performed to assess the differences of road traffic accident mortality in Guizhou with the whole nation. The regional differences between Eastern, Central and Western areas of China were also compared. Analysis of variance was used to analyze the differences in road surface type. Multiple linear regression analysis was used to explore possible influencing factors between the number of deaths and GDP-per-capita, education level, the number of health institutions, population, and car ownership status. All tests were two-tailed, and *P* < 0.05 was considered statistically significant.

## Results

Basic road traffic accident characteristics included a total of 2,261,126 traffic accidents, 2,528,225 persons injured, and 648,678 fatalities. Resulting direct economic loss from accidents totaled 10661.76 million RMB in China during 2007–2016 (Table 1).

**Figure 1:**
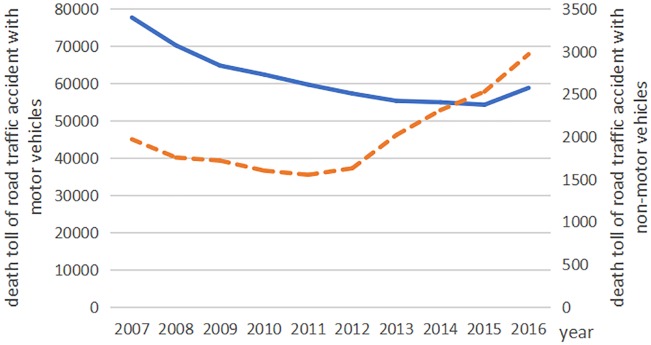
The road traffic accident death toll with motor vehicles and non-motor vehicles in China from 2007 to 2016. The blue line indicates the road traffic accident death toll with motor vehicles and the orange dotted line indicates the road traffic accident death toll with non-vehicles.

**Figure 2:**
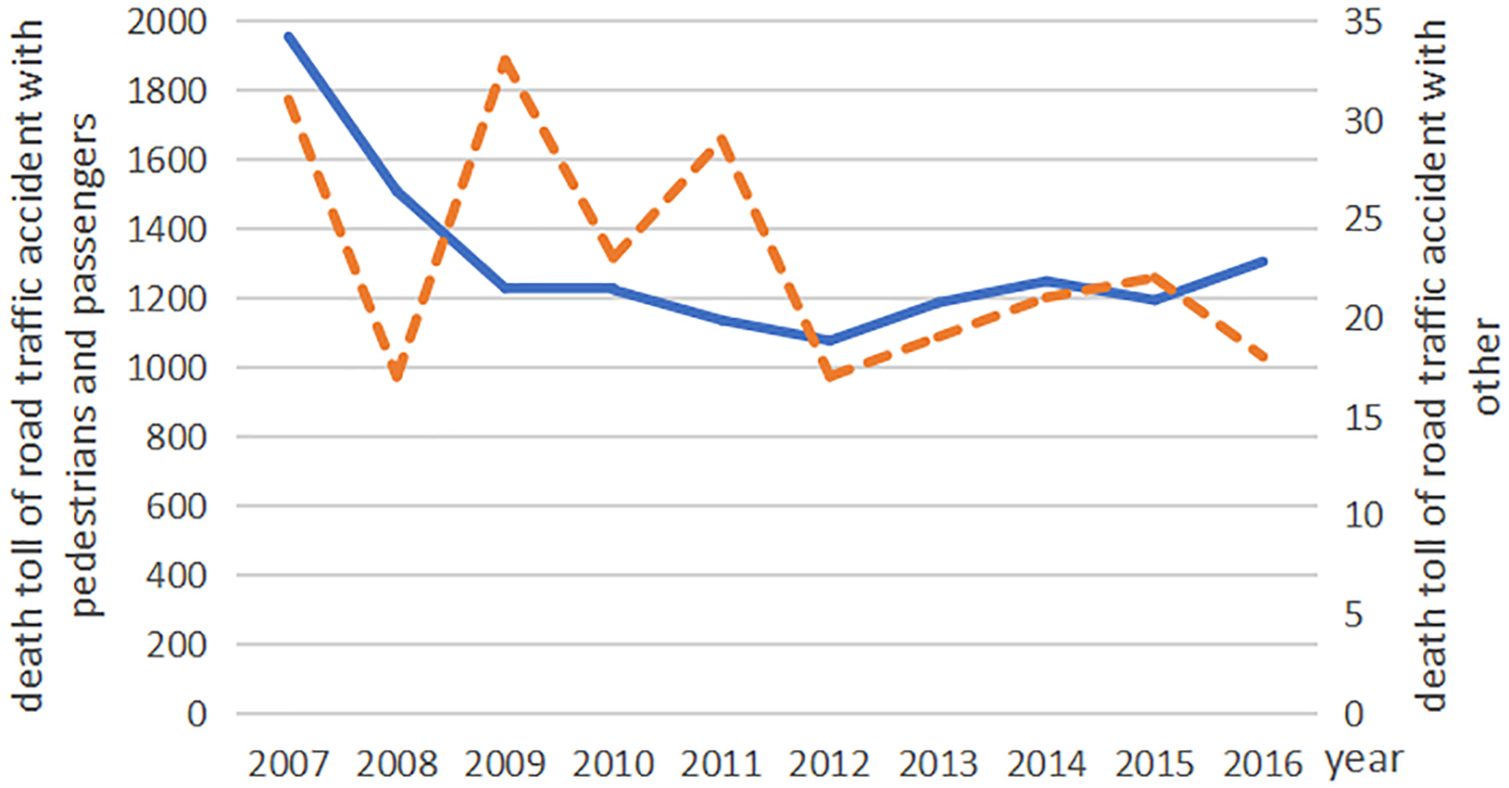
The road traffic accident death toll involving pedestrians and passengers compared to other accidents in China from 2007 to 2016. The blue line indicates the road traffic accident death toll with pedestrians/passengers and the orange dotted line indicates the road traffic accident death toll with other traffic accidents.

**Figure 3:**
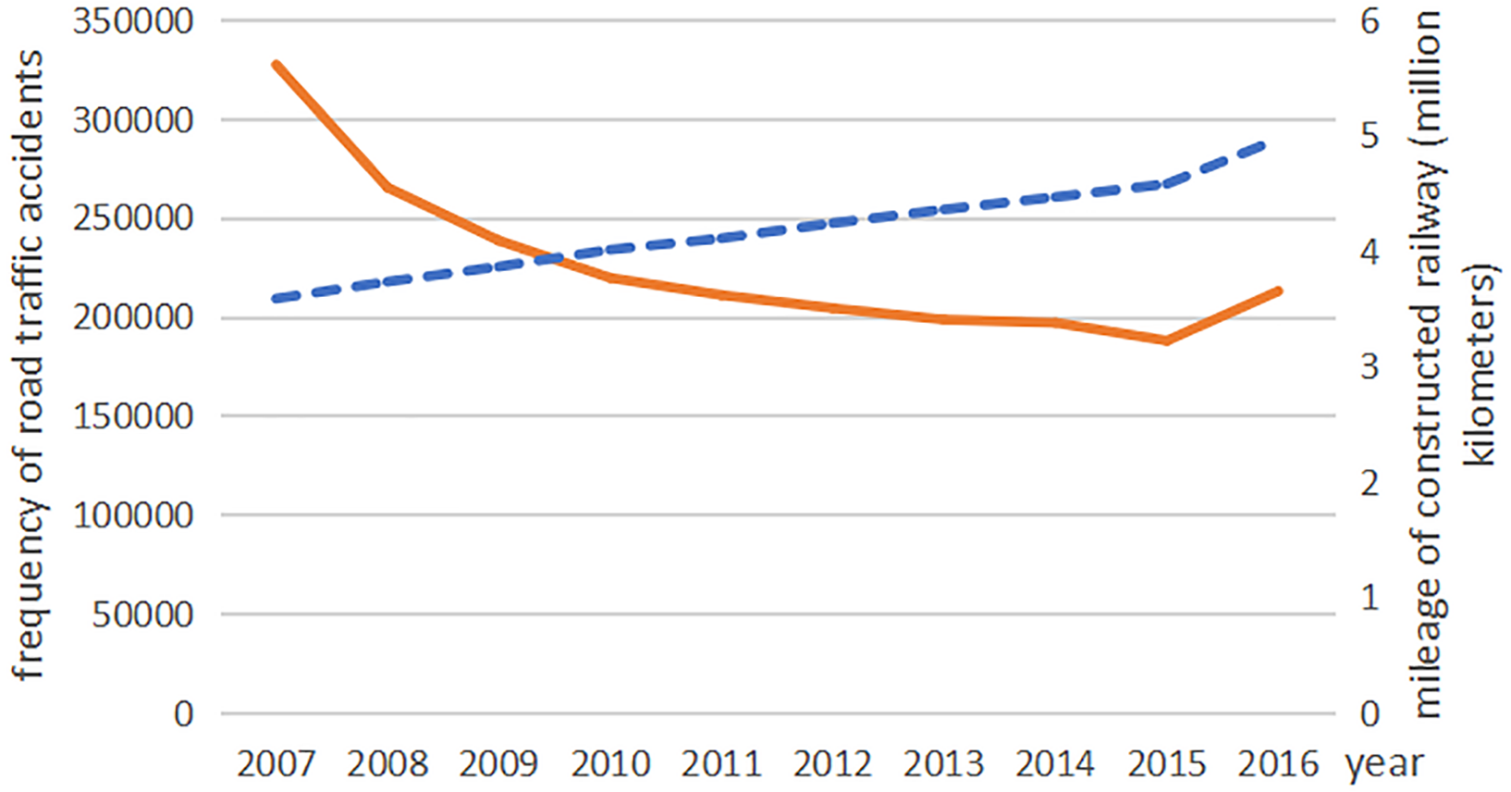
The frequency of road traffic accidents and the mileage of constructed railway s in China from 2007 to 2016. The blue dotted line indicates the mileage of constructed railway and the orange line indicates the frequency of road traffic accidents.

The death toll from road traffic accidents with motor vehicles declined from 2007 to 2015, but rose again in 2016. The deaths from non-motor vehicle accidents declined from 2007 to 2011; however, it rose again rapidly since 2012 (Fig. 1). In recent years, the number of deaths from traffic accidents involving pedestrians and passengers has remained relatively stable, but there was great change in the trends of the ‘other’ traffic category (Fig. 2). From 2007 to 2015, the frequency of road traffic accidents declined despite an increase in the total mileage of constructed highways (Fig. 3).

**Table 1:** Characteristics related to road traffic accidents in China from 2007 to 2016.

Year	Frequency	Death toll	Injured people	Economic loss (Million RMB Yuan)
2007	327209	81649	380442	1198.78
2008	265204	73484	304919	1009.72
2009	238351	67759	275125	914.37
2010	219521	65225	254075	926.34
2011	210812	62387	237421	1078.73
2012	204196	59997	224327	1174.90
2013	198394	58539	213724	1038.97
2014	196812	58523	211882	1075.43
2015	187781	58022	199880	1036.92
2016	212846	63093	226430	1207.60
Total	2261126	648678	2528225	10661.76

From 2007 to 2016, a total of 648,678 individuals died from road traffic accidents in China. The total population in the same years was 13511.98 million, showing a mortality rate of 4.8 per 100,000 people. The Chi-square test showed lower mortality rates in Guizhou compared with the whole nation, and there were statistically significant differences between Eastern, Central and Western areas of China (all *P* < 0.001). Approximately 50% of fatalities of road traffic accidents occurred in the Eastern region, 27.17% occurred in the Western region and 24.73% occurred in the Central region of China. Comparisons between any two areas were statistically significant (*P* < 0.001) (Table 2).

**Table 2:** Comparison of traffic accident mortality rates in different areas from 2007 to 2016.

Group	N (%)	Mortality rate/ 100,000 population	*χ*^2^**	*P* value**
China	648678 (100.00)	4.80		
Guizhou	11658 (1.80)	3.30	1332.74	<0.001^a^
Eastern areas	311991 (48.10)	5.64		
Central areas	160409 (24.73)	3.77		
Western areas	176278 (27.17)	4.82	896.17	<0.001
				
East vs. Central			724.29	<0.001
East vs. West			234.30	<0.001
Central vs. West			369.27	<0.001

**Notes.**

a Mortality rate, Guizhou province vs. the whole nation (China).

Moreover, correlations were calculated between the above factors and the number of deaths (dependent variable). The minimum value of the correlation coefficient between independent variables (GDP-per-capita, education level, the number of health institutions, population, and car ownership status) was 0.83, which indicates that the above five factors had significant correlation with traffic accident death tolls, and thus create a feasible multiple linear regression model (Table 3). The results of the multiple linear regression model and the variable assignments based on quartiles are shown in Table 4. In addition, the determination coefficients of the regression model were relatively high (*R*^2^ = 0.94, *F* = 1511709.75, *P* < 0.001) which indicated that the regression model fit the data well (Table 4).

**Table 3:** Correlation coefficients related to fatalities of traffic accidents.

Independent variables	Correlation coefficient	*P* value
GDP-per-capita (*X*_1_,Yuan)	0.87	0.000
Education level (*X*_2_, 10^4^ persons)	0.83	0.000
Health institutions(*X*_3_)	0.84	0.000
Population (*X*_4_, 10^4^ persons)	0.86	0.000
Car ownership (*X*_5_, 10^4^ vehicles)	0.95	0.000

**Table 4:** The regression coefficient value of each factor with fatalities of traffic accidents.

Variables name	Symbol^a^	B	Standard error	Standardized coefficient	*t*	*P*
(Constant)		454269.58	3234.71		140.44	0.000
GDP-per-capita (Yuan)	X_1_(1 to 4)	0.39	0.00	0.53	92.97	0.000
Education level (10^4^ persons)	X_2_(1 to 4)	−10.85	0.06	−0.34	−187.30	0.000
Health institutions	X_3_(1 to 4)	0.10	0.00	0.38	213.52	0.000
Population (10^4^ persons)	X_4_(1 to 4)	−2.84	0.02	−0.67	−129.29	0.000
Car ownership (10^4^vehicles)	X_5_(1 to 4)	−50.68	0.04	−1.51	−1168.03	0.000
Death tolls (persons)	Y (Observed original value)					

**Notes.**

a Based on the quartiles of each variable.(1=“ <P_25_”, 2=“P_25_-”, 3=“P_50_-”, 4 ≥P_75_). P_*x*_ is percentile.

The equation of the regression model could be obtained by the above coefficient values:

\begin{eqnarray*}Y=454269.58+0.39{X}_{1}-10.85{X}_{2}+0.10{X}_{3}-2.84{X}_{4}-50.68{X}_{5}+e \end{eqnarray*}

In the regression model, *Y* was death toll, *X*_1_** was GDP-per-capita, *X*_2_** was education level /10^4^ persons, *X*_3_** was number of health institutions, *X*_4_** was population /10^4^persons, *X*_5_** was car ownership status /10^4^ vehicles, and ‘e’ was the residual.

**Figure 4:**
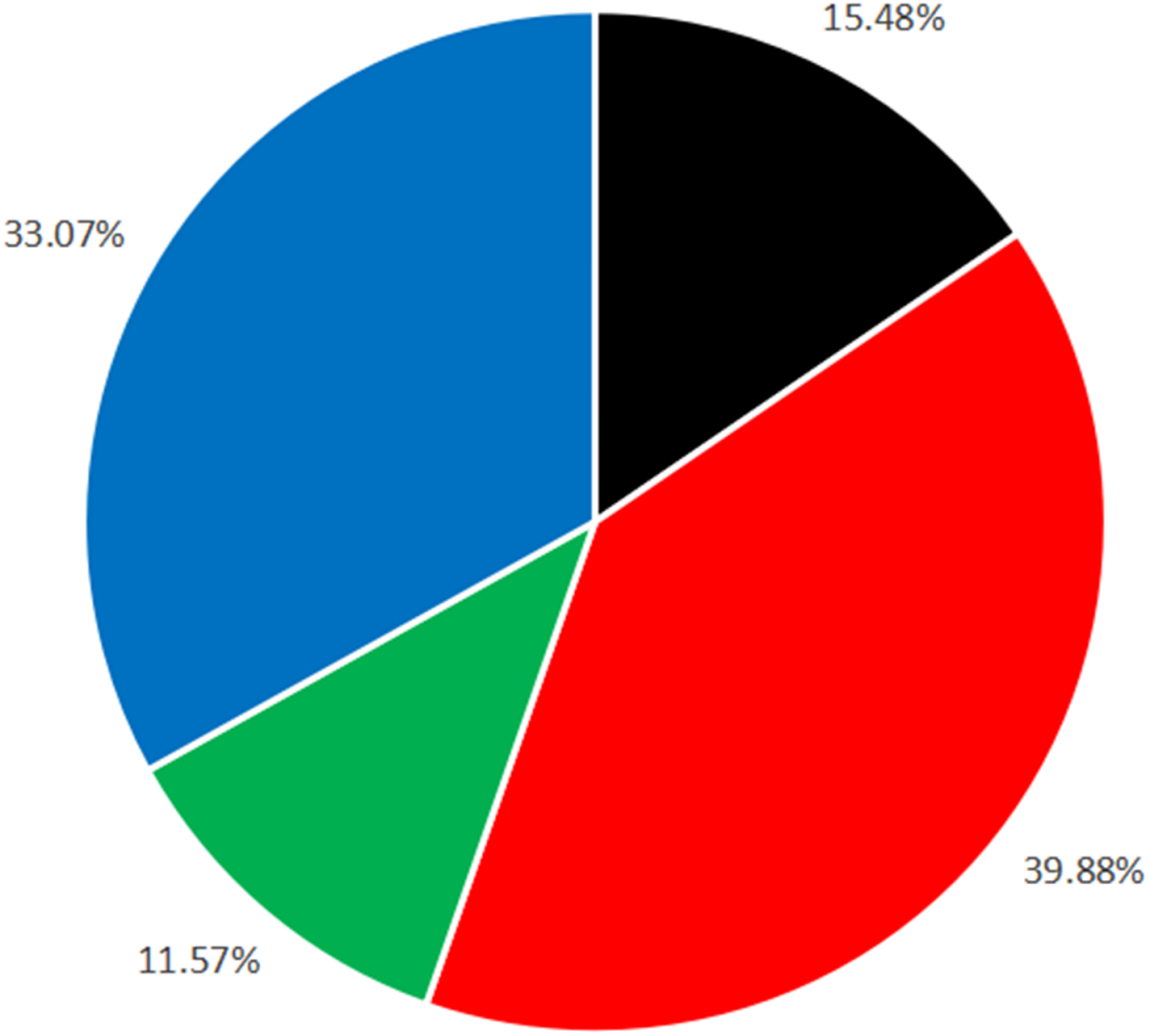
The occurrence rates of traffic accidents on different road surface types. Each percentage shows the occurrence rate of traffic acciddent. The black part indicates the rate of asphalt concrete pavement, the red part indicates the rate of cement pavement, the green part indicates the rate of simple pavement and the blue part indicates the rate of unpaved road.

We found that from 2010 to 2015, the highest occurrence of traffic accidents was on cement concrete pavement roads (39.88%), and this was followed by unpavement roads (33.07%) (Fig. 4). Multiple comparisons showed that traffic accident occurrences were significantly different on different road surface types (all *P* < 0.05) (Table 5).

**Table 5:** Multiple comparisons of traffic accident occurrence between different road surface types.

Road types	Mean difference	Standard error	*P* value	95% Confidence Interval	
				Lower Bound	Lower Bound
1 vs. 2	−49526.67	5118.08	0.000	−68788.28	−30265.05
1 vs. 3	7925.67	1806.88	0.033	728.47	15122.86
1 vs. 4	−35707.83	2660.88	0.000	−44425.80	−26989.87
2 vs. 3	57452.33	4823.82	0.000	37362.52	77542.14
2 vs. 4	13818.83	5204.30	0.188	−5349.97	32987.64
3 vs. 4	−43633.50	2038.32	0.000	−51839.23	−35427.77

**Notes.**

a Road types: (1= asphalt concrete pavement, 2= cement concrete pavement, 3= simple pavement, 4= unpaved roads).

## Discussion

It was a longitudinal study on RTIs in China. Since 2007, road traffic accident frequency had generally declined in China, except for it increased slightly in 2016. It may be due to the government support approval, and the proportion of the State’s investment in both transport construction and management network system had increased, and the road traffic related improvements have displayed initially (Sun et al., 2019).

Road traffic accidents have caused a great hospitalization burden in China. Our results showed that the death toll related to motor vehicles, pedestrians/ passengers declined , and there was large fluctuation in the trends of the ‘other’ traffic category (The actual value was small). Previous literature mainly focus on motor vehicle traffic rather than non-motor vehicles. However, one of notable findings from our study is that non-motor vehicle accidents (such as those involving bicycles, tricycles, electric bicycles, disabled people’s motor wheel chairs and powered vehicles) has risen at a high speed since 2012, which indicates more attention should be given to address this issue in the future. Non-motor vehicles, as a vulnerable group in the traffic system, are rarely paid enough attention to. Non-motorized transportation is an important of urban transportation in China. The reasons for non-motor vehicle traffic accidents increased year by year are as follows: Firstly, the number of non-motor vehicles has increased fast as the advantages of small volume, convenient, flexible, environmental protection and low carbon popular with travelers. However, the road space was more crowded. Secondly, more non-motorized lanes or mixed carriageways for motor and non-motor vehicles are occupied for parking in roads, which further increases the risk of non-motorized vehicles being exposed to motor traffic. Thirdly, non-motorized cyclists, especially e-bike riders, have not been well managed in overtaking, overweight, speeding and other traffic violations (Wang et al., 2017). A simulation study based on health and police data indicated that China was still at a stage of high road traffic mortality (Huang et al., 2016).Our study described a large amount of road traffic mortality. In response, government departments should make more efforts to incorporate safety into road design, enhance road traffic management, and change unsafe behaviors of pedestrians, drivers and passengers to reduce the threats of injury or death.

According to 2009 report, mortality rate in Eastern region was significantly higher than that of Western region and Central region (Zhang et al., 2013), but the data were only one year, possibly can not reflect their difference and trend in a long-term. Our study found that there are wide variations in mortality rates among Eastern, Central and Western over the past decade. It was reported that the number of traffic accidents is positively related to the GDP-per-capita in Algeria in the short and long term, which implies that higher economic development worsens the road safety situation. Moreover, the results suggest that reduced economic activity has been a beneficial impact on traffic fatalities (Bougueroua & Carnis, 2016; Noland & Zhou, 2017). Many road traffic crashes in low- and middle-income countries, where urbanization and motorization are rapidly increasing, may be due to the growing number of motor vehicles on the road (Feng et al., 2014). The Eastern region of China maintains the highest economic growth rates and the number of car ownership registrations was 5,480.71 million during 2007–2016. In the same years, data from the China National Bureau of Statistics showed Central and Western regions had only 3,698.78 million and 3,376.23 million car ownership registrations, respectively. The degree of motorization was evidently higher in the Eastern region than that in Central and Western regions. Thus, we considered that the different economic conditions was an important reason which caused different mortality rates between Eastern, Central and Western areas. Besides that, the traffic system is complex in the eastern plain area. While in the central and western areas, the terrain is complex, and natural disasters such as debris flow and landslide occur from time to time. These are also important reasons for different mortality rates in Eastern, Central and Western areas.

Previous studies also reported that a correlation between vehicle ownership growth and the number of road traffic fatalities and injuries exists (Jiang et al., 2017). In China’s Western region, the terrain with curved roadways (incidence rate ratio (IRR): 1.20, 95% CI [1.08–1.33]) was significantly and positively associated with the number of the extremely serious road accidents (ESRAs) (Liu et al., 2018). Guizhou province is one of the Southwestern regions of China. In Guizhou there are many mountains, rivers, bridges, tunnels and terrain with high altitude. In China, more than 75% of fatal and serious injury crashes are reported to have occurred in mountainous areas between 2007 and 2013 (Chen et al., 2016), so the characteristic and mortality rates of road traffic accidents were different from the whole nation. Therefore, all departments of transportation should take measures according to local conditions.

A road traffic system is mainly composed of humans, vehicles, roads, and the environment. Each subsystem contains multiple influencing factors. If one or more factors deteriorate, traffic safety is compromised and the probability of traffic accidents increases. Our study planned to select broad indicator factors that influence RTIs. We found that GDP-per-capita, education level, the number of health institutions, population, and car ownership status had significant correlations with traffic death tolls, which also indicated that the regression model fit the data very well. Previous studies also reported that GDP-per-capita had a significant correlation with traffic death tolls (Feng et al., 2014; Bougueroua & Carnis, 2016; Tan et al., 2014a; Tan et al., 2014b). Education level and the number of vehicles involved in the accident strongly affected the non-occurrence rate of traffic accidents, which was similar to this study’s results (Tan et al., 2014a; Tan et al., 2014b Oralhan & Goktolga, 2018). Moreover, the dramatic increased in vehicle ownership over the past few years has resulted in an increase in traffic accidents. A previous study also showed road traffic injury mortality appeared to be statistically related to GDP, motorization, population and car ownership (Feng et al., 2014). This study also found that with the increase of the number of health institutions, death tolls showed a downward trend. Therefore, the investment of health institutions needs to be improved (Sun et al., 2019).

Regarding road surfaces, differences were observed between paved and unpaved roads. A study found 12% of their road traffic accidents on unpaved surfaces (Mcgreevy et al., 2014). In our results, the RTI occurrence rate of unpaved roads was higher than simple pavement and asphalt pavement. Many mandatory vehicle signs and safety warning signs can reduce the risks of paved but not unpaved roads (Denning & Jennissen, 2016). There are more unpredictable factors on unpaved roads, and it might cause higher RTI rates than other road surfaces. Safety warnings should explicitly state the dangers of roadway riding regardless of surface type. Vehicle warnings should specifically mention the risks not only on paved roads but also on unpaved roads.

In the previous literature, there are seldom reports of traffic accidents on cement concrete pavement and asphalt pavement. However, another notable finding from our study was that the accident rate of traffic on cement pavement and asphalt pavement were different, compared with other road surface types. The results showed that the risk factors of road type and road surface wetness have an impact on the RTI occurrence of young and elderly drivers with a history of traffic violations, and head-on collisions were more likely on wet surfaces (Olabarria et al., 2015; Feng et al., 2016). In our results, the highest RTI occurrence rate was 39.88% on cement concrete pavement roads. Cement paved roads have an impenetrable porous structure with low absorption ability. Rainwater or other water easily stays on cement concrete pavement surface, and the risk to become wet and slippery increases. This risk causes differences between RTIs on cement concrete pavements and asphalt concrete pavements, as well as differences between cement concrete pavements and simple pavements. We suggest utilizing the porous structure and absorption ability of asphalt concrete pavement to create dry road surfaces. Another study indicates a statistically significant relationship between pavement macrotexture and a logarithm for RTIs. The coefficient for relating pavement macrotexture to RTIs is negative, which means that the number of crashes decreases as pavement macrotexture increases (Pulugurtha, Kusam & Patel, 2010). There is abundant macrotexture on asphalt concrete pavements. Because of those characteristics, RTI occurrence rates on asphalt concrete pavements differ from RTIs on other surfaces.

There are some limitations in our study. Under-reporting biases are a possibility in China; the rate of death from RTIs based on death registration data was about twice as high as the rate reported by the police between 2002 and 2007 (Hu, Baker & Baker, 2011). As our data came from police traffic accident records, under-reporting might be a major study limitation. Second, because the data were collected in a retrospective cross-sectional study, we could not calculate road traffic incidence and mortality rates ourselves. Third, detailed information of road traffic accident frequency on different road surface types was not available from the police registry database, and this data was obtained through evaluating according to the proportion (Total highway mileage / Total traffic accident frequency = Highway mileage of a kind of road pavement / x). Due to these limitations, our study mainly provided results about some changing trends and possible influential factors of road traffic accidents in China.

In summary, our study indicates that the different characteristics of RTIs among the different regions, road surface types and possible influential factors in China. Thus, further interventions might be focused on the targeted influential factors, in high-risk regions and road surface types. Moreover, future studies should consider how to conduct some targeted interventions and assess their effects to reduce the RTIs in China.

## Conclusion

It is noteworthy that in our study, the increasing trend of the death toll related to non-motor vehicles and the highest occurrence on cement concrete pavement roads. Our findings suggested subsequent research could explore the relationship between deaths with non-motor vehicles and cement concrete pavement roads, such as the mechanisms, occurring law, preventive measures; and further clarify the independent risk factors of RTIs. Moreover, the decision-makers and planners in China can draw advantage of the findings for giving some target preventative measures to reduce RTIs, such as learning the efficient traffic safety management methods from the other countries to control RTIs.

##  Supplemental Information

10.7717/peerj.7423/supp-1 Supplemental Information 1 Total data for road traffic injuries

## Additional Information and Declarations

### Data Availability

The following information was supplied regarding data availability: The raw measurements are available as a Supplemental File.
